# Nurture together: trauma-informed and culturally safe perinatal support for migrant mothers

**DOI:** 10.3389/fgwh.2026.1690896

**Published:** 2026-03-30

**Authors:** Anna Beesley, Lucy Lowe

**Affiliations:** Social Anthropology, University of Edinburgh, Edinburgh, United Kingdom

**Keywords:** hostile environment, migrant women, migration and health, perinatal care, refugee health, trauma and maternity care, UK immigration system

## Abstract

British immigration policies are intended to create a “hostile environment” for “undesirable” migrants within the UK and a deterrence to anyone beyond its borders. The seeping of these policies into everyday life, including healthcare provision, for all migrants has been well documented. This article draws on ethnographic research with migrant women who have precarious immigration statuses in Glasgow, which explored interwoven experiences of maternal health, migration and motherhood. Building on this research, the authors co-developed “Nurture Together”, a participatory project with migrant women and Amma Birth Companions, which created trauma-informed and culturally safe peer support resources for infant feeding. Drawing on both projects, this paper highlights how the hostile environment and the subsequent isolation, discrimination, and fear of unfamiliar social and medical environments can compound the trauma experienced by many women during and after migration to the UK. We emphasise the importance of communities of care for mothers who are not surrounded by their usual perinatal support networks.

## Introduction

Economic and racial disparities in maternity experiences and outcomes in the UK are well documented, notably in higher maternal and infant mortality rates for Black and Asian people and for those living in the most deprived areas (1). For migrant women, this is further complicated by a policy and legislative landscape that has fostered confusion and discrimination surrounding rights and access to services. This paper examines the perinatal experiences of women with insecure immigration statuses living in the context of the British immigration system, commonly referred to as the “Hostile Environment”. We examine how this environment, which encourages surveillance, penalization, and removal, impacts women's experiences of seeking care.

A systematic review of perinatal health outcomes and care among asylum seekers and refugees indicated that migrant women generally have poorer health outcomes, notably maternal mortality, mental health, and preterm birth (2). Refugee and asylum-seeking women are at greater risk of experiencing maternal mental distress than the general population (3). Violence and trauma are common reasons for migration and displacement, and women often experience trauma during their migratory journeys (4). Human trafficking and exploitation also cause and contribute to an array of physical and mental health issues (5). This is particularly pertinent for women who have experienced sexual violence and exploitation, which can include forced pregnancies and abortions, as well as sexually transmitted infections and physical trauma (71). Women with experience of trafficking and exploitation have been found to have higher rates of mental health problems (72, 73). The gendered impact of sexual violence includes pregnancy and the difficulty involved in bonding with a child born from rape (6: 37–8). Women's experiences of trauma can be compounded after their arrival in the UK by experiences of racism, isolation, anti-immigrant discrimination (7), as well as the asylum process itself (4, 8, 9).

Migrants in the UK encounter many obstacles to accessing healthcare. There are initial visa checks and Immigration Health Surcharges for specific migration routes, overseen by health professionals performing “borderwork” (10). Language barriers, staff and patient confusion regarding healthcare entitlements, and the widespread fear and stress of living with an insecure immigration status can all cause delays in accessing perinatal care (11). Moreover, the globally persistent stigma surrounding mental health can exacerbate the barriers to accessing appropriate care, particularly for women migrating from countries where mental healthcare is not widely available (12). However, regular routine antenatal appointments present an opportunity for signs of mental distress to be identified and addressed.

Migrant women in the UK also experience divergences in the quality of care they receive during and after pregnancy. A scoping review of quantitative research on intrapartum clinical interventions among people with experience of the asylum system in high income countries found that refugee and asylum-seeking women were less likely to receive pain relief (13). However, the same study highlighted that the data on induction and augmentation of labour and rates of caesarean sections was inconsistent, reflecting the lack of homogeneity among “refugees and asylum seekers” as a defined group (13). Nationality, racialization, socioeconomic status, and other intersecting axes inform perinatal experiences and outcomes, as do the social, political, and economic context of the host country and community. A systematic review of perinatal care for asylum seeking and refugee women demonstrated similar challenges to those experienced by other migrant women, but with heightened challenges of racism, discrimination, stigma, stereotyping and language barriers from perinatal healthcare services and professionals (2). Women with different immigration statuses (e.g., asylum seeker and student) from the same country might have more comparable experiences than asylum seekers from different countries. Similarly, anti-black racism might result in some comparative experiences between Black British women and Black migrant women.

“Migrant women” constitutes a heterogenous group, with differences in nationality, race, language, immigration status, and class, among others, significantly impacting perinatal experiences and outcomes. Grouping together such diverse populations and experiences risks obscuring important differences and inequalities, and perpetuating the promotion of inadequate and potentially ineffective broadbrush policies and interventions (2). This qualitative project, however, was designed to allow participants to express their individual experiences, shaped by a myriad of intersecting factors, while collaboratively sharing and analyzing their experiences as migrant women. Furthermore, the intersectional approach of this project highlights that experiences of pregnancy and birth cannot be defined by singular factors in isolation, such as race, migration status, or class. Instead, it is essential to examine how these are entangled, and can result in commonalities and divergences across and within categories.

The participants in this research reflect this heterogeneity and include people in the asylum system, those with refugee status or other forms of protection, people who had experienced trafficking and exploitation, and people with spousal (dependant) or student visas. Although diverse in their immigration statuses, they shared some crucial experiences as racialised women from African and Asian countries who were or had been pregnant and/or were caring for their babies in Glasgow, Scotland, and had experiences of National Health Service (NHS) maternity care and health visitor support. Exploring where these intersectional experiences converge can reveal the impact of the “hostile” immigration environment and perinatal care on migrant women.

Glasgow provides a useful site for examining perinatal support for migrant women. In addition to a long history as a city of immigration and emigration, Glasgow was the only asylum dispersal city^1^ in Scotland from its inception in 1999 until 2022, and at the time of writing it remains the largest in the UK, outside of London. The number of people with active asylum claims fluctuates, but during this research there were around 4,000–5,000 in Glasgow (14). The number of people in Glasgow with refugee status or other forms of humanitarian protection, and those with other types of insecure immigration statuses, such as students or people with spousal visas, are not available. This legacy has generated an array of support services, charities, and community-based organisations that actively shape perinatal and migratory experiences.

In order to understand the contexts of our participants' lives, we begin with an overview of the UK immigration and asylum environment and the perinatal landscape, before unpacking and discussing the key themes that emerged from our data.

### Immigration and asylum in the UK

#### The Hostile environment

The “Hostile Environment” was fostered by legislation and policies of control and punitive surveillance that have seeped into the fabric of everyday life (15). It centres on the notion of belonging, dividing those who do and do not have a right to call the UK “home”, and is inextricably linked to ideas of deservingness. The birth of the Hostile Environment is widely attributed to then-Home Secretary Theresa May, who stated in 2012 that the aim was to make life as difficult as possible for people unable to provide evidence of their right to be in the UK. However, this was far from a radical shift in British immigration policy, but instead reflected the proliferation of anti-immigration measures that escalated across the twentieth and into the twenty-first century (16).

Central to the creation of the Hostile Environment, argue Griffiths and Yeo (17), is the “deputization” of border enforcement to third parties. The Immigration Acts of 2014 and 2016 enhanced the restrictions and surveillance on everyday activities including rental accommodation, obtaining bank accounts or driving licenses, and accessing healthcare (18: 28). These Acts significantly expanded internal bordering processes in the UK, shifting bordering from the edge of nation-states into everyday life. Rather than geographical lines, borders are processes and “borderwork” (10) is now conducted by state institutions, organisations, private companies, and members of the public.

The National Health Service (NHS) can be analysed as a “bordering scape” (19, 20). The *National Health Service (Charges to Overseas Visitors) Regulations (2015)* introduced new treatment fees for overseas visitors and routine immigration checks for patients conducted by hospital and community healthcare staff. The data sharing agreement, through NHS Digital and Health and Social Care, allows the Home Office to obtain non-clinical data on patients whose immigration status they deem suspect (17). These implements create moral and ethical dilemmas for healthcare professionals who might be required to turn away patients based on their immigration status, yet are further complicated by the insufficient knowledge and training they receive to make decisions of entitlement (21). Ultimately, it disrupts the healthcare professional-patient relationship, negatively impacting both parties, in particular vulnerable groups that are discouraged from accessing timely care (22).

The frequent lack of awareness among many NHS staff and other border workers of the legal or social ramifications of the hostile environment reinforces the marginalisation of people seeking asylum and other migrants. As a result, people working in the voluntary sector can feel like they carry the burden of “bridging the gap” to accessing mainstream services. Balaam et al. (23) found that voluntary sector workers in the north of England, who had a more comprehensive and holistic understanding of the experiences of pregnant refugee and asylum seeking women before and after their arrival in the UK, were well placed to understand how maternity services could be adapted to better meet their needs.

The Hostile Environment seeps throughout wider society, creating a culture of suspicion (24) whilst increasing, and arguably validating, anti-immigration sentiment and racism. It is a system, argues Goodfellow, “whereby it was deemed acceptable to treat people not as human beings but as problems” (15: 6). The recent *Nationality and Borders Act (2022)* and the *Illegal Migration Act (2023)* have entrenched the perception of immigration as an urgent political problem, and have intensified efforts to prevent people from entering the UK, limit access to basic rights and services, and facilitate swifter detention and deportation. This negative, burdensome and criminal image of refugees and other migrants portrayed by many politicians and the media have contributed to everyday experiences of racism. Far-right riots and demonstrations took place across the UK during this research, including widespread anti-immigrant and racist violence in the summer of 2024.

This pervasive hostility intentionally fosters a sense of constant surveillance for people with insecure immigration statuses, leading to a persistent fear of punishment, detention, or deportation. Yet the Hostile Environment's “technology of control” reaches well beyond the undocumented migrants that the British Home Office claimed to be their target, “breed[ing] suspicion, fear and sensitisation of the boundaries between those who belong and those who do not” (25: 18), including migrants with lawful immigration status and racialised British citizens (9, 15). As a result, many people with precarious immigration statuses experience acute social isolation. Maguire (26) suggests that such loneliness should be understood as structurally enforced by the hostile environment, rather than an accidental outcome. The compounding effects of isolation, exclusion, and fear often prevent people from accessing much needed healthcare, including maternity care (17). It is therefore unsurprising that restrictive immigration policies are associated with poor health outcomes (27). Importantly, the consequences of these policies are also highly gendered and experienced differently across different intersectional groups (6, 70).

#### Immigration status

Pregnant migrants are particularly stigmatised because gendered and racialised discourses on reproduction are fundamental to NHS bordering (20). Pregnant migrants' access to maternity care is limited both because they are seen as “temporary” and therefore not “at home” and that, by having a baby in the UK's NHS, they are making a claim to a home (20: 142). The cessation of British jus soli citizenship in the 1981 Nationality Act formalised the perception of migrant women's fertility as a threat to the nation state by curtailing their capacity to produce desirable future citizens. In addition to being a threat, providing maternity services to these migrants is also viewed as economically non-productive. A “key moral legitimation strategy” is exempting the most vulnerable from NHS bordering, for instance asylum seekers, refugees, and victims of human trafficking (20: 151).

Our research participants had differing immigration statuses, and in some cases their status changed during the course of the research, for example, from asylum seeker to refugee status. Immigration statuses determine which services individuals can access and whether or not they are required to pay for them. People seeking asylum are not allowed to work, except in limited circumstances, and have no recourse to public funds (NRPF). Those who are destitute and awaiting a decision are entitled to asylum support in the form of no-choice accommodation and very limited financial support from the Home Office. Asylum seekers are entitled to access NHS care.

If an asylum claim is successful, the applicant will be granted refugee status. In some cases, the Home Office decides that an applicant does not meet the narrow criteria for refugee status but does qualify for humanitarian protection because they would still be at risk of harm if returned to their country of origin. At the time of writing, people with refugee status or humanitarian protection are granted five years' leave to remain in the UK, after which they can apply for Indefinite Leave to Remain. They are allowed to work, access NHS services, and apply for benefits.

If someone is refused asylum, the Home Office will terminate support and ask the applicant to leave the UK. People with children under the age of 18 will still be entitled to financial support. Pregnant people in the asylum system or refused asylum who are in receipt of Section 4 support are entitled to a maternity grant of £300 and a small increase to their regular financial support (28). Those with small children will also receive extra payments to support their children.

Everyone in the UK is entitled to NHS maternity care, however some people are required to pay for such care due to their immigration status (28). Those entitled to free NHS maternity care include refugees and people with humanitarian protection, people in the asylum system, and those in Scotland, Wales, or Northern Ireland who have been refused asylum, or in England who have been refused asylum and are in receipt of Section 4 or Local Authority support, people who have been identified as victims of modern slavery, people in immigration detention, and people who paid the health surcharge when applying for their visa. However, rules around charging for care are complex which has resulted in asylum seekers being wrongly charged (29), creating further distress and uncertainty around accessing healthcare.

People with a temporary right to be in the UK, such as students, usually have a visa stipulation of No Recourse to Public Funds (NRPF). Some people with NRPF status can access limited grants, such as the Best Start Grant in Scotland (Healthy Start Scheme in England, Wales and Northern Ireland) which provides pregnant women and parents of young children with financial help to purchase healthy food, milk and free vitamins.

The Children Act (1989) states that there is a legal requirement for local authorities in the UK to provide for children, regardless of immigration status, which means that people with NRPF might be able to access limited support and accommodation, for example through social work services. However, this is distinct from mainstream social security benefits. Nonetheless, the financial, emotional, and logistical pressures of raising children can exacerbate the challenges of surviving and navigating an asylum and immigration system designed to make life difficult and cause destitution (18).

As childcare falls disproportionately to women, and in the case of this research, often single mothers, the violence of the hostile environment is highly gendered. This gendering is also evident at the intersection of precarious immigration and domestic violence (6). The majority of people in the UK with a dependant's visa in the UK are women (30), which can leave them vulnerable to abuse. Their NRPF status means that they cannot access many support services or refuges that are publicly funded, which can leave people trapped in relationships that are vital to their ongoing right to remain in the UK.

#### The UK asylum process

Not all the women we worked with had been through the asylum process but for those who had it had a significant impact on their wellbeing and their mothering journey. No-choice accommodation and very limited financial support in addition to the complexities of the often-lengthy asylum process can exacerbate the impact of previous harms and trauma, perpetuating oppression and structural violence (6, 9, 31).

The “initial accommodation” used for asylum seekers including hotels, military barracks, and ferries, has been widely criticised and deemed unsafe and unsuitable for pregnant women and those caring for any children under the age of 18 (32–34). These facilities are often catered with fixed mealtimes, and therefore can be missed, and limited food options, which can prevent pregnant women and new mothers from accessing healthy and appropriate food (35). Moreover, financial support is extremely low. At the time of this research it was £8.86 per person per week when housed in catered accommodation (36, Asylum Support).

The 1999 Immigration Act introduced a no-choice dispersal system for asylum applicants in receipt of government support. This system, justified as a means to relieve the “burden” of the number of applicants residing in London and Southeast England, disperses applicants to designated cities across the UK (37). Dispersal, along with detention and deportation, has been described as the state's “arsenal of control” in the ongoing attempt to manage undesirable migration (38).

Dispersal is a way to exclude people going through the asylum process, moving them away from social networks, kin ties and communities across the UK at very short notice. This can happen repeatedly and without any justification or choice in the often-lengthy period before an asylum decision is reached. No-choice movement of accommodation does not only happen across cities and countries in the UK, but also within cities themselves. Although this is a smaller geographical move, it can still be experienced as further displacement, with disruptions to housing, local support systems, and community spaces that are often essential to people's wellbeing. Asylum seekers are often housed in areas of multiple deprivation and frequently in poor and/or unsuitable homes. This can result in existing local residents, who might be waiting for new accommodation themselves, viewing them as being prioritized and therefore fueling resentment towards the new arrivals (39: 34).

Furthermore, the impact of poor and/or unsuitable accommodation on pregnant women and mothers with babies and children is well documented. They are often housed in multi-story flats or tenement buildings, without suitable access for prams or space needed for babies and children, such as beds and cots, exacerbating the stress of the perinatal period (28, 34, 40).

The dispersal system leaves people in the asylum process isolated, lonely (26) and in need of re-building a life and community, often more than once. Again, the impact of this is highly gendered as women are at higher risk of exploitation and relocation during the perinatal period can compound stress for mothers.

#### Birth in the UK

Birth outcomes in the UK are stratified. The MBRRACE report (1) on maternal mortality in the UK indicated that Black women are three times more likely, and Asian women are twice as likely to die from pregnancy related causes when compared with white women. Women living in the most deprived areas of the UK have a maternal mortality rate more than twice as high as women living in the least deprived areas (1). The report also highlighted the negative impact on women's health when they experience multiple disadvantages. Most of our participants experience such compounding disadvantages.

Poor maternity outcomes are also reflected in widespread experiences of trauma during childbirth. A 2024 inquiry into birth trauma in the UK found that poor care is “all-too-frequently tolerated as normal” (41:9). It also found that women from marginalized groups, notably racialized minority groups, experienced particularly poor care, with some reporting direct and indirect racism (41:8). Gill Walton, chief executive of the Royal College of Midwives, explicitly blamed “institutional racism” for these outcomes (42).

Disparities in maternity outcomes and experiences must be situated in the context of chronic underfunding and staffing shortages in the NHS, which have contributed to recent concerns regarding the overall safety of maternity services (43). Midwives across the UK have been found to experience high levels of anxiety, depression, work-related stress, and burnout (44). The Royal College of Midwives (45), found three quarters of midwives working in Scotland had considered leaving midwifery, 41% had not been given access to statutory training during work hours, and half of respondents said their hospital or maternity unit “rarely” had safe staffing levels.

Disparities must also be situated within the Hostile Environment and an NHS in which clinical decision-making has become borderwork (22) and pregnant migrants are widely perceived to be economically burdensome (20). Lonergan (19) draws on Shellee Colen's (46) theory of “stratified reproduction” to demonstrate how the policies and practices of the hostile environment, including inadequate housing, dispersal, and the opacity of both the NHS and the Home Office, result in differential distribution of resources and support for reproduction (2024: 237). While Lonergan (19) predominantly focuses on the role of the state and institutions, stratified reproduction also demands attention to wider social perceptions of “desirable” and “undesirable” reproduction and “good” and “bad” mothers. In this vein, Dana-Ain Davis' (47) theory of “uneven reproduction” argues that disparate reproductive outcomes must be understood as the result of “complex patterns of investment and disinvestment that reconfigure reproduction”. Migrant women, and indeed many non-migrant racialised women, are subject to such patterns of social and economic disinvestment that can render their reproduction as problematic and potentially dangerous.

## Methods

Beesley and Lowe conducted 30 months (March 2022 – August 2024) of ethnographic research, qualitative semi-structured interviews, and participatory arts-based research with female asylum seekers, refugees, and people with other precarious immigration status, such as student or dependents visas, in Glasgow. Creative arts-based methods included quilting, textile printing, and zine making. The project was grounded in an feminist ethical research praxis that sought to examine asylum, displacement, and migration through a reproductive justice lens, which recognises the right to have a child, the right to not have a child, and the right to raise children in a safe and healthy environment (48). All the participants we worked with identified as women and mothers and therefore this is the language we use in this paper. However, we acknowledge the lack of inclusivity in this language, and that the needs of trans and non-binary parents have not been captured by this research.

In October 2023 the authors began an additional project, working in collaboration with Amma Birth Companions (*Amma*), a Glasgow-based charity that provides perinatal support to vulnerable people. The project, Nurture Together, sought to develop trauma-informed, culturally sensitive resources to support breastfeeding^2^. We worked with an “Experts by Experience” group of fourteen women with experience of migration, including asylum, trafficking for sexual exploitation, students, and dependents. Most of the group had received support from *Amma* in the form of volunteer doulas to support their birth and/or immediate post-natal period. The women came from six countries in Africa and Asia. This project was guided by a steering panel composed of the authors, *Amma* staff, lactation experts, including a Speciality Breast Doctor, a GP, lactation consultants, and former *Amma* clients with lived experience of seeking asylum in Glasgow. The steering panel offered responsive guidance throughout the project, including reflections on findings and analysis, suggested outputs and resource development, and advice for the researchers on how to approach the potentially sensitive topic of race and racism with a diverse collaborative group.

Beesley and Lowe facilitated 10 workshops with the Experts by Experience group. During these, the group collectively reviewed existing breastfeeding resources, examined the specific challenges and needs of migrant women, received training from lactation experts on supporting breastfeeding and dispelling popular misconceptions about infant feeding, discussed reproductive rights and justice, and collaboratively designed appropriate tools to support breastfeeding parents, guide NHS staff, and advise policy-makers. The ethnographic research was designed to capture the breadth and nuances of migrant women's perinatal and mothering experiences in Glasgow, while Nurture Together aimed to collaboratively produce guidance to improve and support migrant women's infant feeding experiences. Both projects generated data on infant feeding and how they were impacted by birth experiences, accommodation, social security, destitution, and mental health. We draw on data from both strands of research in this article, and we present the findings under the key themes that emerged during the ethnographic research and participatory discussion in the Nurture Together workshops; birth, trauma and mental health; fear; isolation; being a “good” mother; and community.

The long-term ethnography and the participatory workshops allowed participants the time and space to develop relationships with the researchers and to direct the focus of conversations. It also allowed the researchers to consult interlocutors on key themes as they emerged as part of an iterative analysis. The research was conducted in English, with interpreters provided where required. As a result, most women who participated in the long-term ethnographic research were able to converse in English to at least a basic level. The insights of women who spoke very little or no English are therefore mostly limited to interviews and creative arts-based workshops.

Whilst this research involved women of varying nationalities, ages, immigration statuses and stages of motherhood, all of the participants had received support at some point from third sector organisations and none of the participants were undocumented at the time of research. Therefore, the voices of the most marginalised and least connected migrant mothers are potentially missing. Although research was conducted with other stakeholders, including third sector staff and volunteers, the research did not include comprehensive research with NHS staff due to capacity limitations. As a result, the paper does not capture their perspectives on trauma-informed or culturally safe care, or their capacity to deliver them within their current working conditions.

### Ethics

Due to the sensitive and potentially distressing nature of some aspects of this research, an intentionally slow approach was taken to this research in order to give participants sufficient time to consider their participation, ask questions, and develop trust with the researchers. Participants were provided with written and oral information in advance of the research. People seeking asylum often have safety concerns about being identified, and their experiences of persecution can make them uncomfortable or fearful of signing formal documents. Participants were therefore given the option of providing oral or written consent.

### Voice/positionality

Both authors are white, middle-class, cis-gendered British women living in heteronormative family units. Whilst being able to share some experiences of birth, mothering and motherhood with participants, there were crucial differences and power imbalances at play.

The Nurture Together project and resources were co-produced between the researchers, *Amma*, and the participants of the group. However, we must critique the extent to which genuine co-production can take place, particularly as an all-white research team working in this area. Thinking carefully about the statement made by Ifeyinwa et al. when discussing breastfeeding research, “it is unethical and potentially harmful to conduct research on disparities and inequities with all White research teams” (49, 450). With this in mind, the authors took a feminist approach to conducting trauma-informed ethical research (50), including slow and participatory methods to decentre the researchers and allow participants to direct the research as much as possible.

## Findings

Living within the constraints of immigration policies and practices, many of the women involved in this research had to manage with little financial resources, an underlying uncertainty around the right to stay in the UK, and a maternity system in crisis. Participants had diverse experiences of relationships with midwives, health visitors, and doctors, ranging from highly positive and supportive to very negative. Nonetheless, key areas of concern emerged consistently among participants during interviews, quilt-making, conversations over cups of tea, and the Nurture Together workshops.

This section presents participants' experiences of pregnancy and mothering through five themes: birth, trauma and mental health; fear; isolation; being a “good” mother; and community. These themes reflect the larger structural inequalities that participants experienced as precarious migrants. The Hostile Environment policies contributed to everyday feelings of isolation and fear that shaped if and how they engaged with healthcare providers and other support services. Many of the women we worked with felt discriminated against due to their immigration status or racialization. Whilst hostile policies and practices aim to make connections difficult, and the consequences of which can seep into communities of care, most of the participants highlighted the importance of their community and peer support to cope with motherhood in the UK.

### Birth, trauma and mental health

This section describes how migrant mothers’ past experiences and current situations impact their births. The women involved in this research arrived in the UK through varying routes and circumstances, and had different experiences once in the UK including with their perinatal care. However, most participants raised distressing experiences that fell during two main periods; before arrival in the UK and during their perinatal experiences in the UK. Firstly, participants discussed how experiences relating to migration and asylum had negatively impacted their maternity experiences. This included separation from or deaths of children and other family members before arriving in the UK, as well as experiences of sexual and gender-based violence before, during, and after migration. It is well established that women are disproportionately affected by sexual and gendered violence throughout the migration process, and certain groups such as trafficked women even more so (6: 28). As stated above, the UK's asylum system is itself traumatizing and often has a negative impact on the mental and physical health of people seeking refuge (6, 31).

Secondly, women talked about traumatic experiences during the perinatal period, often relating to poor or discriminatory care in NHS settings. Amma Birth Companions (51) highlights the high rates of intervention and experiences of discrimination faced by their clients, 96% of whom were non-British/migrants. We saw similar issues in our research. Women were usually assigned to the “red pathway” of maternity care, which involved more surveillance and more interventions during pregnancy and birth. Whilst there was inconsistency in how people were assigned to different pathways, being an asylum seeker, and therefore deemed a “vulnerable group”^3^, appeared to be a reason to be put on the “red pathway”. During ethnographic research, women would often mention their confusion at the frequency and reasons for their antenatal appointments. Crucially, participants told us that their options and rights were not adequately explained to them, and at times interventions were performed without informed consent.

In 2022, 48% of Amma clients had an induction of labour and 60% had a caesarean birth, which is higher than the already high rates of interventions in birth in Scotland.^4^ Some women who had experienced sexual violence opted for caesareans because they thought it would be less traumatizing than a vaginal birth. Although it is essential that people can make informed decisions, birthing options are not always a choice. Some participants wanted caesareans because they were single mothers with other children at home and no one to look after them. Scheduling a birth allowed them to plan their limited childcare. This was “chosen” despite the physical toll of undergoing major surgery and the concomitant recovery. Nonetheless, the freedom to make decisions is crucial, whilst acknowledging the importance of intersectional analysis when thinking about who gets to choose and how. Although solo parents can get support from social services in the form of emergency foster care in such situations, the women who participated in this research were often terrified that being on the radar of social services would put their children at risk of removal.

Even when women spoke fluent English, they often felt that their medical care was not adequately explained to them. Many women were told they required inductions because their babies were “too big” or “too small” and were often confused when they eventually delivered an average sized baby, often after considerable trauma. Cynthia, a participant from West Africa, explained:

“The midwives were nice and explained the situation, but the doctor was terrible. She really pressured me to have an induction, she kept telling me my baby was at risk, he might die. I eventually agreed to have an induction at 39 weeks, but I went into labor naturally the day before I was due to have the induction. When I went in, they looked at my record and saw I was due to have an induction, so they put me on the Pitocin drip without asking me. I didn't know what it was at the time, I only found out later. They never asked for my consent. And then things went bad, my baby's heart rate was dropping, and they decided I had to have a c-section. They rushed me to theater and I had the anesthesia, but then the doctor tells everyone to stop, I'm 10 cm dilated so they can't do the c-section, I have to push. But I'm numb, I can't feel anything, I can't push, so they have to cut me and use forceps to deliver my baby”.

Cynthia felt out of control and pressured into a birth she did not want. This situation is made even harder when mothers do not speak English. The lack of access to adequate interpretation for migrants in the healthcare setting has been well researched (52–54) and was a recurring problem for Amma clients and our research participants during perinatal medical interactions, including childbirth. In-person interpreters were extremely rare, and midwives frequently struggled for long periods to get an interpreter on the phone, often being left on hold and calls being disconnected. This is distressing for patients and stressful for NHS staff, especially during medical emergencies. All the women we worked with were confident, at the time of the research, to read, write and speak in English. However, Noor, a participant from Afghanistan, explained at the time of her birth her English was “zero, I can't speak but there is no translator”. She had an interpreter during her birth but not in the postnatal ward and therefore could only understand “like 40% of what they told me”. Leaving new parents, particularly mothers with little to no social networks, with only 40% of the information healthcare providers pass on is potentially a very dangerous situation.

#### Fear

Past experiences and birth trauma are compounded in a context of fear. This section explains how the impact of fear can stop mothers asking for support. The Hostile Environment has tangible repercussions in the everyday lives of the women we worked with. Whilst they might not be explicitly aware of policies and procedures that create the Hostile Environment, the unwelcoming atmosphere, both societally and institutionally, is affective. For some participants this led to a lack of trust in institutions and support services, including healthcare providers, at a time when they and their newborns are most vulnerable. Many of the mothers spoke about being fearful of answering questions, asking for support, or informing care providers of problems and worries they had about their living situations and/or their and their children's wellbeing. This was particularly acute in more formal settings, for example antenatal or GP appointments. There was a common perception that giving “bad” or “wrong” answers might result in getting in trouble with the Home Office and/or having their child(ren) removed. Indeed, social services were a frequent topic of conversation amongst participants, and stories of negative experiences with social workers circulated among migrant mothers more widely. These fears were most often expressed by Black African participants. During one workshop, two South Asian participants were surprised to hear about the concerns expressed by four women from West Africa about the threat of child removal in encounters with NHS staff and social services. These disparities reflect Dana-Ain Davis' (55) suggestion that racialized perceptions of Black mothers as high-risk results in more institutional surveillance and intervention than white mothers.

Fear of negative repercussions can prevent people seeking the medical, financial, or social support that they need, and are entitled to, or from being fully open and honest when they access support. Elizabeth, a participant from West Africa, told us:

“You could be in the worst circumstances - maybe your partner does abuse you, maybe you really can’t afford to look after yourself and your child – but you will say everything is fine, just because of the fear of where that information will go”.

The data sharing agreement between the Home Office and the NHS, as mentioned in the introduction, makes these fears a potential reality. Griffiths and Yeo explain, “it is important to remember that these dire impacts are not accidental side effects of the Hostile Environment but are central to its operation” (17: 531). Indeed, everyday borders are effective when overarching feelings of instability and anxiety are generated creating a “chronically insecure and dehumanised, “deportable”, people” [de Genova, 2002 in (17: 531)].

Fear of speaking out lingers into the postnatal ward and beyond. Noor spoke about a friend of hers who had recently had a baby in Glasgow and when pressing the buzzer for help on the postnatal ward had been told by a midwife, “you’re not in a hotel”. This stayed with Noor and resulted in her not having the “courage” to call for help when her turn came. Not feeling able to ask for help on the postnatal ward means that mothers can miss out on vital care immediately after birth, such as support with breastfeeding or help facilitating mother and baby skin-to-skin after a caesarean section (which can help to initiate breastfeeding). Noor explained:

“they didn't ask [about feeding] at the hospital, just on the morning the doctor comes and asks are you feeding well, I couldn't but I said to the doctor “yes I can” because [I was scared], if I'd said no he will be angry or say something or…”

It was not until Noor had a home visit from a midwife two days later that she was taught how to attach her baby onto her breast.

Fear continues once new mothers return home from the hospital. Pregnant people and new parents are often anxious about going out. The increase in anti-immigrant and racist rhetoric across the UK (and Europe) further impacts this fear among the women we worked with. The 2024 race riots, for example, compounded participants' anxieties around leaving their homes and accommodation due to direct experiences of, or hearing stories about, incidents of hate speech and racism [see also (34)]. In addition to severing feelings of safety, belonging, and making it harder to create social networks, fear also resulted in participants missing important appointments, including perinatal care.

#### Isolation

Mothers' past experiences sit alongside their current circumstances of being a new mother, often in new and unknown surroundings. This section considers the gendered isolation that participants experienced. The bureaucracy, opaque processes, waiting and uncertainty involved in asylum (and other immigration) processes left many participants feeling anxious and depressed. Compounded by fear, anxieties around leaving the home are exacerbated when people are moved between accommodations and therefore forced to repeatedly navigate new and unfamiliar surroundings without a local social network and potentially with little to no English. The frequent moves that asylum seekers undergo as well as traumatic migration-related experiences make it difficult for people to establish, then re-establish, communities and relationships of trust. Not all the mothers we worked with were in the asylum process and were therefore not subject to the same level of forced movement; however, many still experienced isolation and difficulties in establishing social connections within their local area. Rona, a participant from Southwest Asia, told us:

“When I first came here, I didn't know anyone, I didn't know much English. My husband came here first, so he knew Glasgow and he was going out to work all day. When I got pregnant, I was too scared to leave the house in case I got lost or something happened. I never saw women like me with babies. It was all old women. The first time I went to *Amma* I was so happy to see other women like me, who were not from Glasgow and they were pregnant or had their babies. Even when we didn't speak the same language, I was just happy to be with them because I didn't feel alone”.

Approximately half of the participants in the Nurture Together group had their partners (all male) with them during their perinatal period. However, this did not always mitigate the isolation and anxiety that they experienced during this time. The gendered isolation female migrants experience when joining their male partner who is already settled in the UK, as well as the changes to family dynamics, has been highlighted in the context of refugee family reunion [(56); see also (9) for a wider discussion of insecure immigration statuses and coupledom].

Furthermore, male partners were often not seen as active participants within prenatal care and birthing spaces. For many of our interlocutors this was a cultural norm, where female relatives such as mothers, mothers-in-law, aunts, and grandmothers would dominate reproductive spaces, practices, and knowledge (57, 58). Participants would often relay stories of their antenatal appointments completely omitting their male partner from the event despite them being present.

One participant requested a birthing companion from *Amma* as she assumed she would receive better support from a relatively unknown female volunteer than her male partner. Unfortunately, due to the stretched nature of third sector support, the charity was unable to provide her with a birth companion, and had to save this resource for someone without a partner or any other support to draw on. Some of the other participants did not have supportive relationships with their male partners. Aarvi, a participant from South Asia, had an arranged marriage and their subsequent challenges to conceive created a particularly difficult relationship in which she felt unsupported. She eventually had two children yet experienced quite extreme feelings of loneliness and postnatal depression, often interrelated (59), after both births.

#### Being a “good” mother

Most mothers experience societal pressure to be, or present as, a “good” mother, which is itself racialised and moralised. This section uses breastfeeding as a lens to examine this pressure in the context of migrant mothers' lives and argues that, however one mothers or feeds their baby, networks of care and support are essential for their wellbeing. For the mothers we worked with, navigating immigration processes, securing safe accommodation and meeting their and their child(ren)'s basic health needs in a system of persistent legal surveillance and threat was a constant stress and worry. Worries which, as mentioned above, participants did not always feel comfortable disclosing to care providers and other mothers

Adding to this was the pressure to be seen as a “good” mother. One way of being seen as a “good” mother is breastfeeding your child, a correlation arising from multiple sources including public health campaigns (60) social forces, networks, communities and cultures. Many participants spoke about their communities' ideas and practices around breastmilk and the ensuing importance of breastfeeding. Elizabeth spoke about her community in West Africa and the belief that breastmilk impacts children's development, which manifests through their personalities:

“[my community] will say that your baby will not behave well, that if you feed your baby with cow milk, goat milk the baby will come out to behave like goat, they will not be wise … in order to protect your child from behaving like an animal you should breastfeed. If they see a child that is not behaving well they are like “you don't behave like someone that sucks your mother's milk””

All the participants came from communities in which there was an expectation that mothers would breastfeed their child. Whilst adding to the pressure to be a “good” mother, these beliefs also meant that they were navigating between cultures in their countries of origin and in the UK. Daraja spoke about having successfully breastfed her two children in Nigeria but when she had her third child in the UK, she only saw mothers using formula milk rather than breastfeeding. This led her to question whether it was her who had been feeding her child in the “wrong” way. Daraja's concerns reflected common assumptions among our participants that services and practices in the UK were inherently better than those they had experienced in their home countries, highlighting the lingering afterlife of colonialism.

Over the course of the workshops, we had many conversations about the absence of breastfeeding in public in Glasgow, which had surprised many of the mothers and made them feel less confident to breastfeed in public. The group were very interested to learn about the *Breastfeeding etc. (Scotland) Act 2005* which makes it an offence to prevent or stop a person who has a child or in charge of a child from feeding them milk in a public place. The knowledge of this piece of legislation appeared to be a significant support for the group to have the confidence to breastfeeding in public.

Nonetheless, many of the mothers we worked with were not able to choose to feed their baby solely on the breast. Due to social and economic circumstances one participant, Hassana, who was from West Africa and a single mother, purposefully used mixed feeding (breastmilk and formula) so that her baby would not get too attached and therefore could be cared for by others while she had to go to study and work. She told us:

“I noticed babies on sole breastmilk they are always attached, if he is attached to me, I won't be free to do anything. [I have] no support…when baby's so attached its stressful, you won't be able to breathe, you won't be able to give him to someone else”.

However one chooses to feed their babies, it requires networks of care and support. Conversations during the workshops regularly highlighted the holistic support this group of women needed, not only to breastfeed but to keep themselves, their babies and children physically, mentally, and emotionally well. The group understood the benefits of breastfeeding being wider than the nutritional value it adds, being important in bonding with their babies, and with attachment and nurturing more generally. For the mothers, good mental health and successful breastfeeding were inextricably linked. Many were concerned that the stress of their insecure immigration status might spread through their breastmilk to their babies. Indeed, evidence suggests that stress can impact lactation and breastfeeding outcomes (61). These concerns complicated the pressure to be a “good” breastfeeding mother.

### Community

This final section considers what mothers do when they do not have their usual communities around them during the perinatal period as a result of displacement and migration. Whilst participants created their own communities of care, these never fully compensated for the hostility of the immigration system.

A popular adage tells us that “it takes a village to raise a child”, but for many people in the UK, that village – of social, emotional, and financial support – is painfully lacking. The absence of support was felt acutely by the participants in this research, some of whom had a partner, but others were solo parents. For many of the mothers, this was exacerbated by frequent relocation due to the dispersal system mentioned above. All of them were separated from wider family networks that might have provided perinatal support in other circumstances. Ifeoma, a participant from West Africa, explained:

“If I were back home, I would have all my family with me. My mum, mother-in-law, sisters, cousins, everyone. They would cook for me, bring me food, do the cleaning, even massage me! I would only see the baby when I need to feed her. Otherwise, I'm resting.”

Nevertheless, participants created new networks of communal care that supported them socially, emotionally and practically. These relationships are even more vital for mothers who experience the effects of bordering and multilayered precarity (9). Much of this network making, certainly that we as researchers were privy to, was done at and through in-person and digital community groups, some targeted at migrant women, others at new mums, and a small number aimed at both. These networks can be understood as resistance to the political project of anti-immigrant bordering does (25), where migrant women can cultivate active citizenship even though they may be excluded from the legal status of citizenship (62).

Community spaces can become “spaces of sanctuary” (9:8) providing vital support for mothers who are experiencing isolation. As Mary, a participant from East Africa described, “I'm always a mum, 24/7, but when I come here, I get a few hours just to be myself and not have to worry about anything”. Mothers can socialize, rest, and share experiences and information concerning everything from breastfeeding and teething to problems with housing and accessing asylum support. Over the ten-week workshop series it was almost tangible how the space became one of comfort and safety where the mothers breastfed openly in ways that worked for them. Ideas, advice and laughter around practices of mothering were increasingly shared freely between the group. These spaces help to reduce the loneliness that is commonly felt in periods of new motherhood (63) and is enforced through asylum and immigration policies and practices (26).

The space in *Amma's* office and the breastfeeding workshops facilitate(d) friendship making among mothers. Several participants went on to form strong networks of support outside of the formalized groups, relying on each other for things such as childcare when admitted to hospital to give birth. Whereas women without these networks were left with no choice but to reluctantly accept support from social services. Some participants organized more long-term childcare support to help each other attend college or work. Networks of care that physically and practically support new mums and babies are needed throughout the perinatal period but are felt acutely in the days and weeks after the baby was born. For instance, participants talked of the practical difficulties of being alone on a post-natal ward. This was made more extreme after a cesarean section, where lifting a baby from its cot was an impossible task in the hours after birth and with the fear of asking for help that many described.

Furthermore, most participants were involved in online networks that spread beyond the physical spaces of community groups and organizational activities. Mothers were in multiple WhatsApp and Facebook groups that allowed them to share advice, emotional support and material resources, part of what Benchekroun (62) calls “strategic mothering”. They passed on buggies, cribs, and clothes that their own children had grown out of, creating a free or low-cost exchange of commodities that would otherwise be very costly. Sharing resources in this way can build and foster friendships but conversely, argues Benchekroun, when information is withheld it can undermine friendships (9: 90).

Like McCarthy and Haith-Cooper (64) found in befriending relationships between migrant mothers, creating community spaces in which mothers feel safe allowed for conversations that might not happen elsewhere. Mothers disclosed struggles to one another that they did not share in other space, such as appointments with Health Visitors. Importantly, spaces that involved people who are going through similar experiences to each other were particularly welcomed by participants. Cynthia explained:

“I went to one breastfeeding support group, and everyone was really nice but they were all white women, and we didn't have the same concerns. They were talking about being tired and problems breastfeeding, which I felt too, but I was more worried about where I was going to live and if I might have to leave the country, but I didn't feel like I could share those fears with that group. When I went to *Amma* and I met other mums like me, it was such a relief. The other mums, they really looked after me. They knew what I was going through”.

Nonetheless, even in what is generally a supportive and safe group of migrant women that we worked with, attitudes and beliefs around deservingness of particular groups and mothers linger. Different visa routes (and conditions attached to them) can cause tension, stigma and gossip among already marginalized groups of people. This includes perceptions and misperceptions around the financial and material resources others have access to, with stigma often reserved for those going through the asylum process (65). These discourses of deservingness, arguably fostered by the Hostile Environment and border policies (66, 67), means that mothers with insecure immigration statuses may act with a certain amount of caution as to how much personal information they share. Many participants shared that they constantly balance the tension between making much needed connections and protecting themselves and their children (9: 88).

## Discussion

The maternity experiences of the migrant women who participated in this research reflect many of the themes identified by the UK birth trauma inquiry (2024). Women reported not feeling listened to or believed, and their questions and needs were dismissed or ignored. For migrant women, these experiences are compounded by insecure immigration status and a hostile environment, as well as racial discrimination, which is experienced by many British women too. Such inequalities and discrimination can prevent women from seeking care when they need it. However, this compounded vulnerability could be avoided. The Nurture Together mums believed that most people, and particularly NHS staff, have limited understanding of their experiences as migrants. Knowledge of the complex trauma that is prevalent in experiences of forced migration and trafficking, they suggested, was particularly deficient. Beyond the crucial impact of their immigration status and the trauma involved in migration, such women also experience significant challenges concerning housing, destitution, discrimination, and social isolation, which profoundly affect their everyday lives including their access to healthcare. However, most healthcare professionals have little or no training on these issues (5). The mothers we worked with call for a greater awareness of how immigration legislation and policies, and their inherent racism and anti-immigrant discrimination, shapes their everyday lives, including their encounters with the NHS during pregnancy, childbirth, and postnatally.

Compassionate, individualized perinatal care is important for everyone, and this is particularly true for vulnerable women, including refugees, people seeking asylum, and others with NRPF status. Such women are often socially isolated and have experiences of trauma. Research on the health needs of survivors of human trafficking emphasize the importance of trauma-informed and culturally appropriate care, highlighting the importance of continuity of care for establishing a trusting relationship with healthcare providers (68). Furthermore, research with survivors of trafficking in England indicated that maternity care presents an opportunity to identify survivors and provide appropriate care and referrals (74).

Although survivors of trafficking have specific needs, this research highlights the commonalities in a vulnerable (and overlapping) population that requires compassionate care and who might be otherwise overlooked. This requires midwives and other healthcare providers to be equipped with the training, knowledge and capacity to identify such women, recognize their needs, and address them appropriately. It also requires a holistic approach to care and effective working relationships across networks, including different NHS teams, third sector organisations, and community groups. Yet, research with NHS professionals indicated that many felt they “lack knowledge and confidence in how to respond appropriately” (69). It is important to contextualize this within the critically underfunded state of the UK's maternity service, where large-scale strategic change needs to be made in order for healthcare professionals to work safely.

In light of these findings, the Nurture Together team designed a toolkit of resources to improve maternity care and experiences for migrant women^5^. The migrant mothers identified three key audiences for these resources: other migrant women; people who provide direct support for migrant women, including NHS staff, third sector organizations, and friends and family; and government stakeholders. The Nurture Together toolkit includes a package of trauma-informed and culturally safe peer support training, a suite of videos for migrant mums and the people who support them, a policy brief, and a ten-step poster calling for improved maternity care for migrant mums. Although these resources were designed by and for migrant women, those involved in their co-creation emphasised that what they call for - respectful care, clear communication, informed decision-making - are the basic needs of all birthing people, regardless of immigration status.

## Data Availability

The datasets presented in this article are not readily available because Ethnographic material is sensitive and therefore not available. Requests to access the datasets should be directed to Lucy Lowe, lucy.lowe@ed.ac.uk.

## Footnotes

^1^In the UK, the dispersal policy was introduced under the Immigration and Asylum Act (1999) to distribute people seeking asylum away from the south of England and across other parts of the UK.

^2^Details of both projects, including the Nurture Together toolkit can be accessed at https://www.sps.ed.ac.uk/research/research-project/MAMAS

^3^ https://rightdecisions.scot.nhs.uk/shared-content/ggc-clinical-guidelines/maternity/antenatal-general/asylum-seekers-and-refugees-in-maternity-care-1023/

^4^Live singleton births across Scotland 2023/24: spontaneous 46.6%, elective c-sec 20.1%, emergency c-sec 21.7%, instrumental 11.6% Available at: https://publichealthscotland.scot/publications/births-in-scotland/births-in-scotland-year-ending-31-march-2024/

^5^ https://www.sps.ed.ac.uk/research/research-project/MAMAS
